# Supplementary material for: Neoadjuvant Intratumoral Immunotherapy with Cowpea Mosaic Virus Induces Local and Systemic Antitumor Efficacy in Canine Mammary Cancer Patients
Source: Cells. 2023 Sep 8;12(18):2241. doi: 10.3390/cells12182241 (PMC10527658; doi:10.3390/cells12182241)
Supplement: Supplementary file 1 [file cells-12-02241-s001.zip › cells-2590768-supplementary.pdf]

Supplemental file

Canine patient recruitment and selection criteria

Female companion patients with at least one mammary nodule with the longest measurement greater than two cm were recruited. This size cut-off is related to a direct correlation between the malignant nature of mammary tumors and the size; a mass smaller than this defined size could result in enrolling patients with benign tumors [1,2]. Inclusion criteria at diagnosis included absence of metastatic dissemination based on thoracic radiography and abdominal ultrasound; no severe infection or ulceration of the mammary target tumor; no chronic life-threatening disease or any systemic disease that could influence the immune system response (such as endocrinopathies, immune-mediated disease, leishmaniasis and ehrlichiosis); no treatment with immunosuppressive drugs; and absence of clinical signs compatible with inflammatory mammary carcinoma (as pain, warmth, erythema). For this trial, we followed the ARRIVE guidelines 2.0 [3] (supplemental file 1), and, whenever possible, we followed the guidelines from the "Human Intratumoral Immunotherapy Expert Recommendations" [4].

Surgical procedures

All incisional biopsies were collected under sedation (medetomidine 10 μg/Kg and methadone 0.3 mg/kg, both intramuscularly). Analgesia was provided after the procedure with tramadol (3 mg/kg orally every 12 h for 4 days). If the histopathological evaluation of the incisional biopsy evidenced a non-malignant epithelial mammary tumor, the patient was removed from the study. One week after the first inoculation (day 6-9; DTx2), a second eCPMV *in situ* vaccination was administered at the same dose in the target tumor. At this time, dogs were not under sedation, and the intratumoral eCPMV inoculation was performed under topical local anesthesia using a tetracaine ointment.

Two weeks after the first eCPMV dose (day 12-17; DSx), canine patients were surgically treated as follows: regional mastectomy was performed when the largest diameter of the injected tumor was smaller than 3 or 5 cm in dogs with a weight lower or greater than 10 kg, respectively, and complete unilateral mastectomy when it was higher than 3 or 5 cm (if dog´s weight was lower or greater than 10 kg, respectively) or if multiple nodules were present along the mammary chain. If nodules were present in both mammary chains, patients initially underwent mastectomy of the chain containing the injected target tumor and, after 3-4 weeks, a second regional/complete mastectomy was performed. Seven dogs presented contralateral mammary nodules and a second mastectomy was recommended to their owners; five dogs underwent the second mastectomy and owners refused surgery on two dogs. If the patient was not spayed at diagnosis, ovariohysterectomy was performed at the time of the first mastectomy. For surgical procedures, all patients received medetomidine and methadone (10 and 300 micrograms/kg, intramuscularly, respectively), followed by induction with propofol (1 mg/kg, intravenously) and inhalational anesthesia with isoflurane (1.5%-2.5%). Intravenous cephazolin was given 20 min before surgery (22 mg/kg). Further, depending on the mastectomy procedure, transversus abdominis plane block with bupivacaine (up to 2 mg/kg) and/or epidural anesthesia using morphine (0.1 mg/kg) plus bupivacaine (up to 2 mg/kg) was provided. Diffusion catheters were placed during surgery (DC Mila International Inc®) in order to administer bupivacaine (1-2 mg/kg every 6h) in the post-operative period. Catheters were left in place for 3 days. Soft sterile wound dressings and a tubular mesh were placed to cover the wound. Post-surgical therapy also included firocoxib (5 mg/kg,

orally every 12 h for 7 days) and tramadol (3 mg/kg, orally every 12 h for 3 days). No post-operative antibiotics were prescribed. The wounds healed uneventfully, and skin sutures were removed after 12 days.

Standard adjuvant therapy

After surgery, metronomic chemotherapy with cyclooxygenase-2 inhibitor (firocoxib, 5 mg/kg/daily/oral) and low-dose cyclophosphamide (12.5 mg/m^2/daily/oral) was recommended for CMC patients with grade II (with high Ki-67) and grade III tumors, and/or when regional lymph node metastatic involvement was diagnosed. For grade II mammary carcinomas with low Ki-67, firocoxib alone was recommended. This adjuvant therapy was maintained for two years unless local recurrence or metastases were observed.

Hematological, biochemical and flow cytometry analyses.

A blood sample (~10 ml) was collected from each patient at D0, DTx2, DSx, and 30 days after surgery (D45) to evaluate hematologic, biochemistry, and peripheral blood mononuclear cells (PBMCs) changes induced by eCPMV immunotherapy. Hematological analyses were performed using a standard hematology analyzer (ADVIA® 120, Siemens Healthcare, Madrid, Spain). The biochemical panel (glucose, creatinine, urea and alanine aminotransferase (ALT)) of each patient was performed using reflection spectrophotometry (Refrovet® Plus, Scil animal care company, Viernhheim, Germany), total proteins using Biuret's colorimetric test (Bradford Diagnostics, Sigma-Aldrich, St. Louis, MO, USA), and albumin using a liquid chemistry technique (Randox Laboratories Ltd., Crumlin, UK). PBMCs were isolated for flow cytometry using the Lymphocyte Isolation Reagent kit per manufacturer's instructions (Ficoll 1.077g/ml solution, Rafer, Zaragoza, Spain). Isolated cells were transferred to a freezing medium (70% RPMI, 20% DMSO and 10% fetal bovine serum), frozen at -80ºC overnight, and then transferred to liquid nitrogen until sample processing. Flow cytometry analysis was performed using a 14-color panel (Table S2) with data collection using LSR Fortessa II (BD Biosciences, San Jose, CA, USA) equipped with four lasers and 16 detectors as we previously described [5]. Flow cytometric analysis was performed using FlowJo software (version 10.7.1; BD Bioscience).

Cytokine measurement

The MILLIPLEX® Canine Cytokine/Chemokine Magnetic Bead Panel was used to measure 13 cytokines in plasma samples as indicted by the manufacturer (Merck Millipore, Burlington, MA, USA): GM-CSF, IFN-γ, KC (CXCL1), IP-10 (CXCL10), IL-2, IL-6, IL-7, IL-8, IL-10, IL-15, IL-18, MCP-1 (CCL2), and TNF-α.

Detection of anti-eCPMV antibodies in canine plasma samples.

Serial plasma samples collected at various times during the trial were used to detect the levels of CPMV-specific IgG titers using an Enzyme-Linked Immunosorbent Assay as described elsewhere [6]. Briefly, 96-well microplates (Nunc™ MaxiSorp™ flat-bottom, Thermo Fisher Scientific) were coated with 1 µg of CPMV in 0.1 M potassium phosphate buffer (KP buffer, pH 7.0) and incubated overnight at 4 °C. Plates were washed three times with washing buffer (0.05% (v/v) Tween-20 in PBS, 300 µl per well) after coating and between all subsequent steps. The plate was then blocked with 200 µl of blocking solution (3% (w/v) BSA, in PBS) and place in a microplate

shaker incubator (37 °C, 1 h). Plasma (1 µl in 200 µl of PBS) samples from canine patients were added to the wells and 1:1000 dilution of rabbit anti-CPMV antibody (0.9 µg/ml; clone PAC 12273/12274; Pacific Immunology, Ramona, CA, USA) was used as a positive control (100 µl/well, 1% (w/v) BSA in PBS). As a negative control, no plasma sample was added to the wells, followed by the addition of 100 µl/well of 1% (w/v) BSA in PBS. Plates were then incubated (37 °C, 1 h) in a microplate shaker incubator. Plates were then incubated (37 °C, 1 h) in a microplate shaker incubator. Next, after wash the plates three times with washing buffer, they were incubated with 1:5000 dilution (100 µl/well, 1% (w/v) BSA in PBS) of horseradish peroxidase-labeled goat anti-canine IgG (H+L) Secondary Antibody (stock concentration of 2 mg/ml, Invitrogen, Thermo Fisher Scientific) and 1:5000 dilution (100 µl/well, 1% (w/v) BSA in PBS) of HRP-labeled goat anti-rabbit IgG (H+L) (stock concentration of 1 mg/mL, Fisher Scientific) for the rabbit anti-CPMV antibody positive control wells, respectively. The wells were developed with 100 µl of 1-Step™ Ultra TMB-ELISA substrate solution (Thermo Fisher Scientific) for 2 min at room temperature and quenched with 50 µl of 2N sulfuric acid (Spectrum Chemical). Absorbance was read at 450 nm using the Infinite 200 Pro® microplate reader and the software i-control™ (Tecan, Männedorf, Switzerland).

Immunohistochemistry (IHC) assays

Single 3 µm tumor tissue sections were used for histopathology and IHC. The IHC assays for Ki67, estrogen receptor, progesterone receptor, human epidermal factor receptor-2 (HER2), myeloperoxidase, CD3, FoxP3, CD20, and MUM1. Deparaffination and antigen retrieval were performed in a PT Lab Vision module (Thermo Fisher Scientific Inc, Waltham, MA, USA) by immersion in 1mM EDTA buffered solution at 95 °C for 20 minutes, the sections were cooled down, and immunolabelled in an automatic autostainer (Autostainer 480S, Thermo Fisher), using a polymer-based method and a peroxidase detection system (UltraVision Quanto MAD-021881QK, Master diagnostic, Granada, Spain). The IHC conditions are described in table S3. The same positive tissue control was used as the negative control slide without the primary antibody.

Scoring of IHC markers

Proliferation index (PI) was established as the percentage of Ki67-positive neoplastic cells in 10 high-power fields (40x). A cut-off of 22% was used to distinguish between high and low Ki67 tumors [7]. Inflammatory cells in the tumor microenvironment were assessed by quantifying the total number of cells per square millimiter (counted in ten 20x fields) that were positive for myeloperoxidase (MPO; neutrophils), CD3 (T lymphocytes), FoxP3 (T regulatory lymphocytes), CD20 (B lymphocytes), and MUM1 (plasma cells). T regulatory lymphocytes/T lymphocyte ratio was calculated dividing the number of FoxP3+ cells/mm^2 by the number of CD3$^+$ cells/mm^2. The estrogen receptor (ER), progesterone receptor (PR) and HER2 receptor status was defined per guidelines described elsewhere [8]. Tumor subtypes were defined as Luminal A (ER$^+$/PR$^+$, HER2$^-$, low PI); Luminal B (ER$^+$/PR$^+$, HER2$^-$, high PI); HER2$^+$ (ER$^-$/PR$^-$, HER2$^+$, high PI), and TN (ER$^-$/PR$^-$/HER2$^-$, high PI). Biomarkers are reported following REMARK guidelines [9].

RNA-seq library preparation and sequencing

Frozen tumors samples were homogenized by bead beating with 2 mm stainless steel beads in Qiagen RLT buffer and RNA extracted using RNeasy Mini kits (Qiagen, Redwood City, CA). RNA was quantified by qubit and quality assessed on a Fragment Analyzer instrument (Agilent,

Santa Clara, CA). All RNA samples used in this study had RNA integrity number scores >7. 3'-end RNAseq libraries were produced from 200 ng RNA using the Quantseq Rev chemistry (Lexogen, Greenland, NH) and pooled for sequencing on an Illumina NextSeq2000, 1x100bp run, targeting a minimum of 10M reads per sample.

RNA-seq analysis

FastQC [10] was used for quality control (QC) of raw sequences. Trimming of adapter sequences and poly-A tails was performed with cutadapt [11]. Trimmed reads were aligned to the *Canis lupus familiaris* genome (RefSeq accession: GCF_000002285.5) using STAR [12]. Raw counts were generated for each gene using the Featurecounts package [13]. Samples from two sequencing runs were batch corrected using combat (v.3.42.0) [14]. Expression levels for each transcript were normalized using DESeq2's median of ratios (v1.20.0) [15]. Differential expression analysis was performed using the DESeq2 package (v1.20.0) [15]. An FDR < 0.1 was used to assign differentially expressed genes (DEGs). Principal component analysis (PCA) was performed on the normalized gene expression matrix. GSEA was performed using the fgsea package (v1.20.0) [16]. The C2 (canonical pathways), C3 (regulatory target gene sets), and C7 (immunologic signature gene sets) gene sets were obtained from the human MSigDB Collection (v2022.1.Hs) [17] and used as query for GSEA. Immune cell infiltration was inferred using CIBERSORTx and the LM22 reference matrix [18]. For GSEA and CIBERSORTx analyses, human orthologs were used as input. By performing these analyses as such, we assumed sufficient homology between canine and human cellular processes to allow for interpretable results.

Statistical analyses

Primary outcomes were efficacy, measured by reduction in Tv in the injected target tumor and in noninjected mammary nodules; biosafety, measured by evaluation of hematological and biochemistry changes in blood and plasma. For evaluation of individual eCPMV-induced changes in tumor size between follow-up days (DTx2 and DSx**)** and start of treatment (D0), linear regression analysis of the percentage of changes in tumor volumes was performed. To evaluate potential toxic and immunological effects of eCPMV therapy in dogs, a two-tailed Student's t-test or, as appropriate, Wilcoxon test were performed to compare eCPMV-induced changes in blood cell numbers, plasma levels of total proteins (albumin and globulins), glucose, urea, creatinine, and ALT, and cytokine levels in samples collected before, during treatment, at surgery, and 1 month after surgical procedure. Individual changes in blood parameters were analyzed by linear regression analysis. The immunolabeling of IHC markers were compared between pre-treatment and post-treatment biopsies to determine the effect of eCPMV on the tumor tissue using paired t-test or Wilcoxon test, as appropriate. Pearson's correlation coefficient was used to evaluate the correlation between continuous variables. Two-tailed P values less than 0.05 were considered statistically significant. Statistical analyses were carried out using IBM SPSS Statistics program (version v.25; Armonk, NY, USA) and GraphPad Prism (version 7.02; GraphPad San Diego, CA, USA) software.

Supplementary figures and tables.

Figure S1. Mammary tumors on P1 at D0. Target tumor located in second left mammary gland (blue), non-target tumors on ipsilateral mammary chain (P1.1, P1.2 & P1.3) located in cranial part of second left, medial aspect of third left and medial aspect of fourth left mammary glands (red), and non-target tumors on contralateral chain (P1.4, P1.5 & P1.6) located in third, fourth and fifth right mammary glands (green).

Figure S2. eCPMV immunotherapy does not affect red blood cell count and hemoglobin levels during treatment period. Changes induced by eCPMV injections in the hematocrit (A) and hemoglobin (B). Each companion dog is represented by a colored individual shape as indicated on the right side of B. X-axis indicates the day when measurements were taken. Y-axis shows percentage changes in red blood cells (A) and hemoglobin levels (B). NR, refers to normal range values. *, P-value obtained by paired Student t-test.

Figure S3. eCPMV therapy does not affect hepatic, renal and digestive functions, and immune response in the vaccinated dogs. Changes induced by eCPMV injections in proteins (albumin and globulins; A), glucose (B), creatinine (C), urea (D), and ALT (E) levels. Each companion dog is represented by a colored individual shape as indicated on the right side of B. X-axis indicates the day when measurements were taken. Y-axis shows the plasma concentration of the different biochemical biomarkers. NR, refers to normal range values. *, P-value obtained by paired Student t-test.

Figure S4. eCPMV immunotherapy induced changes in different peripheral blood immune cells. Changes induced by eCPMV treatment in lymphocytes (A), monocytes (B), mature neutrophils (C) and immature neutrophils (D) of individual CMC patients. Each companion dog is represented by a colored individual shape as indicated on the right side of B. X-axis indicates the day when measurements were taken. Y-axis indicates the number of different immune cells per microliter. NR, refers to normal range values. *, P-value obtained by paired Student t-test or Wilcoxon test.

Figure S5. Gating strategy for immunophenotyping of canine PBMCs. (A) Leukocytes (SSC-A/FSC-A) were further defined as single (doublet exclusion FSC-H/FSC-A) live cells (SSC-A/Viability Aqua). (B) Live leukocytes were further discriminated in CD45+ leukocytes and CD14+CD45+ monocytes. (C) Differential expression of MHCII and CD4 on monocytic population. (D) CD45+ leukocytes were divided into lymphocytes (CD22+ B cells and CD5+ T

cells) and (E) a CD22-CD5- cell population further characterized into MHCII+ antigen presenting cells and CD4+ neutrophils ([1] and references therein). (F) Gating for GzmB+CD3- NK cells. (G) Identification of CD4+ T helper and CD8+ T cytotoxic cells within the CD5+ T cell population, and FoxP3+ regulatory T cells differentially expressing CD25 within the CD4+ T cell population (H). (I) Expression of cytotoxic cell marker GzmB within the CD8+ T cell population. Parent population is indicated above the plots. Description of the immunophenotype is provided in table S10.

Figure S6. eCPMV-induced changes in blood immune cells. Changes induced by eCPMV treatment in $CD8^+$ T cells (A), $CD8^+$Granzyme B^+ T cells (B), regulatory T cells (C), monocytes (D), and $MHCII^+CD4^+$ (E), $MHCII^-CD4^+$ (F), and $MHCII^+CD4^-$ (G) monocytes of individual CMC patients. Each companion dog is represented by a colored individual shape as indicated on the right side of C. X-axis indicates the day when measurements were taken. Y-axis indicates the percentages of different immune cells. NR, refers to normal range values. *, P-value obtained by paired Student t-test or Wilcoxon test.

Figure S7. eCPMV immunotherapy induced transitory changes in plasma cytokines in vaccinated dogs. Changes induced by eCPMV injections in (A) IL-2, (B) IL-6, (C) IL-7, (D) MCP-1, and (E) IL-10 plasma levels. Each companion dog is represented by a colored individual shape as indicated on the right side of B. X-axis indicates the day when measurements were taken. Y-axis shows percentage changes in the plasma concentration of different cytokines. P-value obtained by paired Student t-test.

Figure S8. eCPMV immunotherapy induced anti-CPMV antibodies. (A) The levels of anti-CPMV antibodies in five CMC patients increased after injection of eCPMV nanoparticles and remained higher than D0 at various time points. A few dogs were followed beyond surgery day. Each companion dog is represented by a colored individual shape. Absorbance units at 450 nm is on the y-axis and days after eCPMV treatment in the x-axis.

Figure S9. eCPMV immunotherapy induced changes in the TME. (A) Comparison of PC1 values between D0 and D12-17 samples. P-value calculated by unpaired Wilcoxon signed-rank test. (B) Top 10 transcription factor genes significantly upregulated and 10 top transcription factor target genes downregulated on the pathway level by GSEA comparing D12-17 to D0 samples. (C)

Top 10 upregulated and 10 downregulated pathways from the immune-related pathway gene set (C7; MSigDB) GSEA comparing D12-17 to D0 samples.

Figure S10. eCPMV immunotherapy increases intratumoral inflammatory cells in patient 7. Representative immunostaining of pre-treatment (D0) and post-treatment (D12-17) tumor tissues. When compared with pre-treatment tumor biopsies, post-treatment tumor biopsies have significant increases in intratumor infiltration with neutrophils (MPO+), T lymphocytes (CD3+), B lymphocytes (CD20+), T regulatory lymphocytes (FoxP3+), and plasma cells (MUM1+). Quantitative analysis is provided in table S13.

Table S1. Breed of eCPMV-treated CMC patients.

Table S2. List of monoclonal antibodies used for flow cytometry.

Table S3. List of primary antibodies used for immunohistochemistry.

Table S4. Tumor changes in target injected lesion in CMC patients by itRECIST criteria.

Table S5. Tumor changes in target noninjected lesions in CMC patients by itRECIST criteria.

Table S6. Tumor changes in nontarget noninjected lesions in CMC patients by itRECIST criteria.

Table S7. Blood cell and biochemistry changes during eCPMV immunotherapy in CMC patients

Table S8. eCPMV-induced changes in peripheral PMBCs.

Table S9. eCPMV-induced changes in plasma cytokines levels.

Table S10. DEGs comparing D12-17 to D0. Genes with FRD<0.25 are included.

Table S11. Immunohistochemistry changes induced by eCPMV immunotherapy in injected tumors.

References:

1. Burrai, G.P.; Gabrieli, A.; Moccia, V.; Zappulli, V.; Porcellato, I.; Brachelente, C.; Pirino, S.; Polinas, M.; Antuofermo, E. A Statistical Analysis of Risk Factors and Biological Behavior in Canine Mammary Tumors: A Multicenter Study. *Anim. Open Access J. MDPI* **2020**, *10*, 1687, doi:10.3390/ani10091687.
2. Sorenmo, K.U.; Kristiansen, V.M.; Cofone, M.A.; Shofer, F.S.; Breen, A.-M.; Langeland, M.; Mongil, C.M.; Grondahl, A.M.; Teige, J.; Goldschmidt, M.H. Canine Mammary Gland Tumours; a Histological Continuum from Benign to Malignant; Clinical and

Histopathological Evidence. *Vet. Comp. Oncol.* **2009**, *7*, 162–172, doi:10.1111/j.1476-5829.2009.00184.x.
3. Percie du Sert, N.; Hurst, V.; Ahluwalia, A.; Alam, S.; Avey, M.T.; Baker, M.; Browne, W.J.; Clark, A.; Cuthill, I.C.; Dirnagl, U.; et al. The ARRIVE Guidelines 2.0: Updated Guidelines for Reporting Animal Research. *PLoS Biol.* **2020**, *18*, e3000410, doi:10.1371/journal.pbio.3000410.
4. Marabelle, A.; Andtbacka, R.; Harrington, K.; Melero, I.; Leidner, R.; de Baere, T.; Robert, C.; Ascierto, P.A.; Baurain, J.-F.; Imperiale, M.; et al. Starting the Fight in the Tumor: Expert Recommendations for the Development of Human Intratumoral Immunotherapy (HIT-IT). *Ann. Oncol. Off. J. Eur. Soc. Med. Oncol.* **2018**, *29*, 2163–2174, doi:10.1093/annonc/mdy423.
5. Pantelyushin, S.; Ranninger, E.; Bettschart-Wolfensberger, R.; Vom Berg, J. OMIP-065: Dog Immunophenotyping and T-Cell Activity Evaluation with a 14-Color Panel. *Cytom. Part J. Int. Soc. Anal. Cytol.* **2020**, *97*, 1024–1027, doi:10.1002/cyto.a.24168.
6. Shukla, S.; Wang, C.; Beiss, V.; Steinmetz, N.F. Antibody Response against Cowpea Mosaic Viral Nanoparticles Improves In Situ Vaccine Efficacy in Ovarian Cancer. *ACS Nano* **2020**, *14*, 2994–3003, doi:10.1021/acsnano.9b07865.
7. Carvalho, M.I.; Pires, I.; Prada, J.; Lobo, L.; Queiroga, F.L. Ki-67 and PCNA Expression in Canine Mammary Tumors and Adjacent Nonneoplastic Mammary Glands: Prognostic Impact by a Multivariate Survival Analysis. *Vet. Pathol.* **2016**, *53*, 1138–1146, doi:10.1177/0300985816646429.
8. Peña, L.; Gama, A.; Goldschmidt, M.H.; Abadie, J.; Benazzi, C.; Castagnaro, M.; Díez, L.; Gärtner, F.; Hellmén, E.; Kiupel, M.; et al. Canine Mammary Tumors: A Review and Consensus of Standard Guidelines on Epithelial and Myoepithelial Phenotype Markers, HER2, and Hormone Receptor Assessment Using Immunohistochemistry. *Vet. Pathol.* **2014**, *51*, 127–145, doi:10.1177/0300985813509388.
9. Sauerbrei, W.; Taube, S.E.; McShane, L.M.; Cavenagh, M.M.; Altman, D.G. Reporting Recommendations for Tumor Marker Prognostic Studies (REMARK): An Abridged Explanation and Elaboration. *J. Natl. Cancer Inst.* **2018**, *110*, 803–811, doi:10.1093/jnci/djy088.
10. Babraham Bioinformatics - FastQC A Quality Control Tool for High Throughput Sequence Data Available online: https://www.bioinformatics.babraham.ac.uk/projects/fastqc/.
11. Martin, M. Cutadapt Removes Adapter Sequences from High-Throughput Sequencing Reads. *EMBnet.journal* **2011**, *17*, 10–12, doi:10.14806/ej.17.1.200.
12. Dobin, A.; Davis, C.A.; Schlesinger, F.; Drenkow, J.; Zaleski, C.; Jha, S.; Batut, P.; Chaisson, M.; Gingeras, T.R. STAR: Ultrafast Universal RNA-Seq Aligner. *Bioinforma. Oxf. Engl.* **2013**, *29*, 15–21, doi:10.1093/bioinformatics/bts635.
13. Liao, Y.; Smyth, G.K.; Shi, W. FeatureCounts: An Efficient General Purpose Program for Assigning Sequence Reads to Genomic Features. *Bioinforma. Oxf. Engl.* **2014**, *30*, 923–930, doi:10.1093/bioinformatics/btt656.
14. Leek, J.T.; Johnson, W.E.; Parker, H.S.; Jaffe, A.E.; Storey, J.D. The Sva Package for Removing Batch Effects and Other Unwanted Variation in High-Throughput Experiments. *Bioinforma. Oxf. Engl.* **2012**, *28*, 882–883, doi:10.1093/bioinformatics/bts034.
15. Anders, S.; Huber, W. Differential Expression Analysis for Sequence Count Data. *Genome Biol.* **2010**, *11*, R106, doi:10.1186/gb-2010-11-10-r106.

16. Korotkevich, G.; Sukhov, V.; Budin, N.; Shpak, B.; Artyomov, M.; Sergushichev, A. Fast Gene Set Enrichment Analysis | BioRxiv Available online: https://www.biorxiv.org/content/10.1101/060012v3.
17. Subramanian, A.; Tamayo, P.; Mootha, V.K.; Mukherjee, S.; Ebert, B.L.; Gillette, M.A.; Paulovich, A.; Pomeroy, S.L.; Golub, T.R.; Lander, E.S.; et al. Gene Set Enrichment Analysis: A Knowledge-Based Approach for Interpreting Genome-Wide Expression Profiles. *Proc. Natl. Acad. Sci. U. S. A.* **2005**, *102*, 15545–15550, doi:10.1073/pnas.0506580102.
18. Newman, A.M.; Steen, C.B.; Liu, C.L.; Gentles, A.J.; Chaudhuri, A.A.; Scherer, F.; Khodadoust, M.S.; Esfahani, M.S.; Luca, B.A.; Steiner, D.; et al. Determining Cell Type Abundance and Expression from Bulk Tissues with Digital Cytometry. *Nat. Biotechnol.* **2019**, *37*, 773–782, doi:10.1038/s41587-019-0114-2.

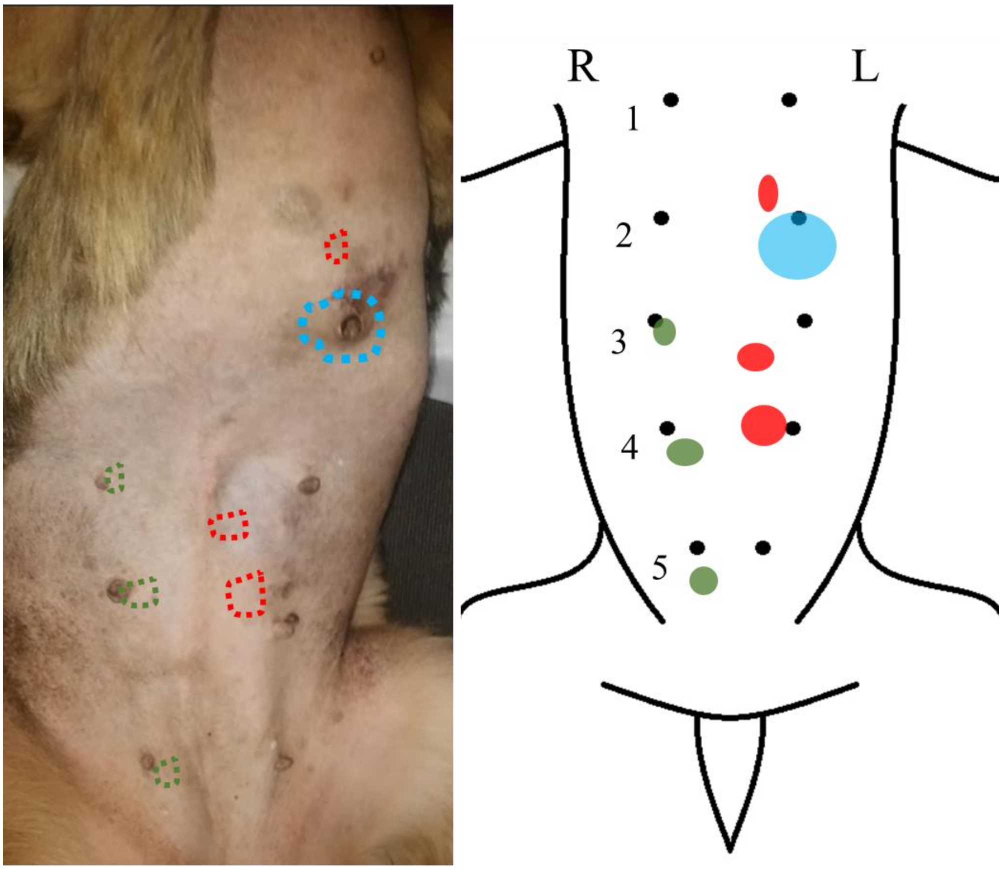

Figure S1. Mammary tumors on P1 at D0. Target tumor located in second left mammary gland (blue), non-target tumors on ipsilateral mammary chain (P1.1, P1.2 & P1.3) located in cranial part of second left, medial aspect of third left and medial aspect of fourth left mammary glands (red), and non-target tumors on contralateral chain (P1.4, P1.5 & P1.6) located in third, fourth and fifth right mammary glands (green).

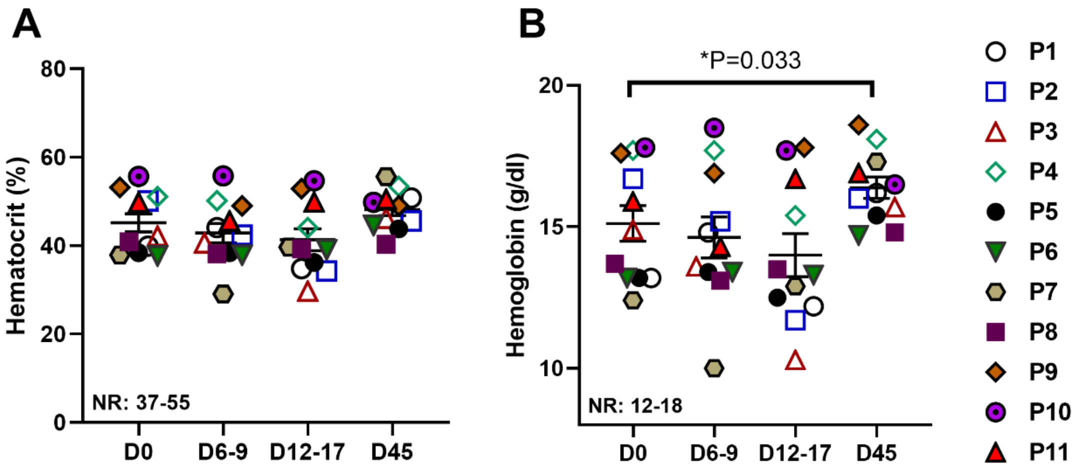

Figure S2. eCPMV immunotherapy does not affect red blood cell count and hemoglobin levels during treatment period. Changes induced by eCPMV injections in the hematocrit (A) and hemoglobin (B). Each companion dog is represented by a colored individual shape as indicated on the right side of B. X-axis indicates the day when measurements were taken. Y-axis shows percentage changes in red blood cells (A) and hemoglobin levels (B). NR, refers to normal range values. *, P-value obtained by paired Student t-test.

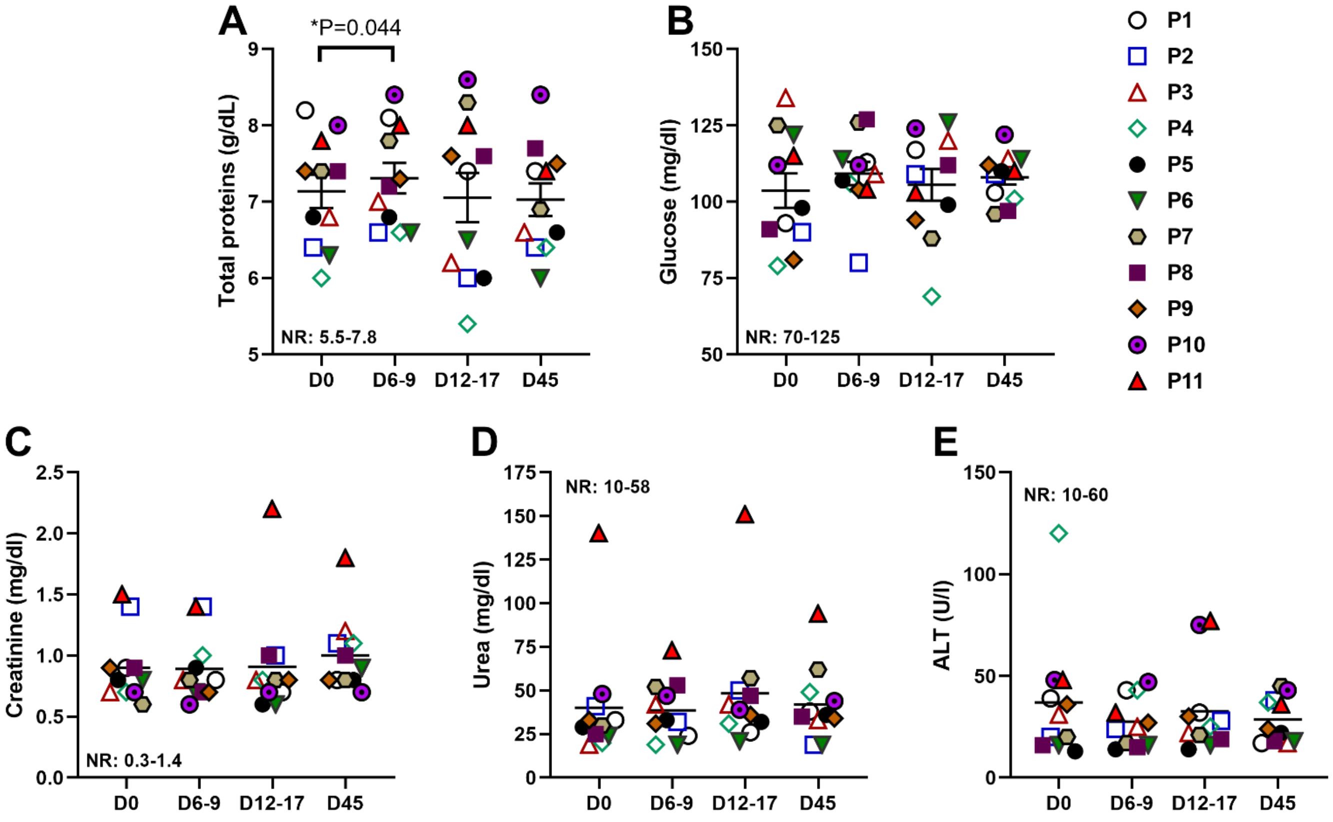

Figure S3. eCPMV therapy does not affect hepatic, renal and digestive functions, and immune response in the vaccinated dogs. Changes induced by eCPMV injections in proteins (albumin and globulins; A), glucose (B), creatinine (C), urea (D), and ALT (E) levels. Each companion dog is represented by a colored individual shape as indicated on the right side of B. X-axis indicates the day when measurements were taken. Y-axis shows the plasma concentration of the different biochemical biomarkers. NR, refers to normal range values. *, P-value obtained by paired Student t-test.

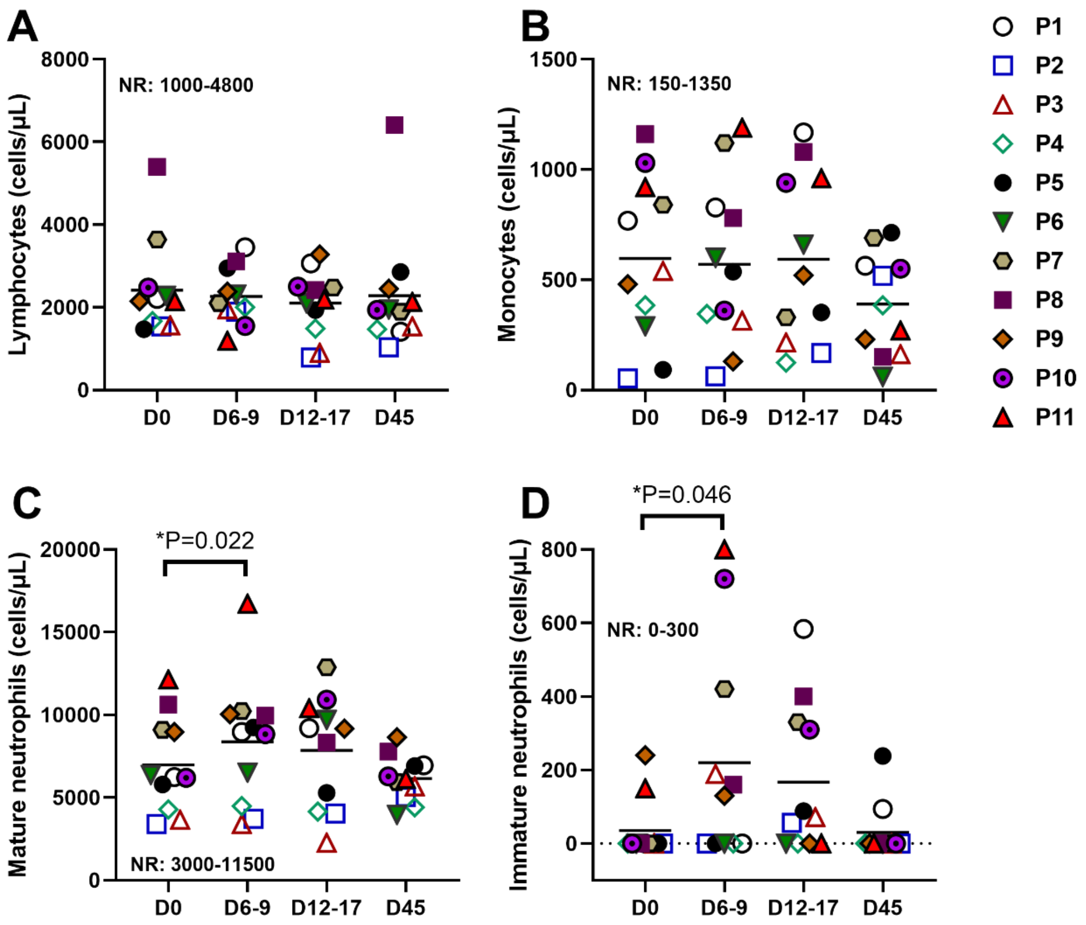

Figure S4. eCPMV immunotherapy induced changes in different peripheral blood immune cells. Changes induced by eCPMV treatment in lymphocytes (A), monocytes (B), mature neutrophils (C) and immature neutrophils (D) of individual CMC patients. Each companion dog is represented by a colored individual shape as indicated on the right side of B. X-axis indicates the day when measurements were taken. Y-axis indicates the number of different immune cells per microliter. NR, refers to normal range values. *, P-value obtained by paired Student t-test or Wilcoxon test.

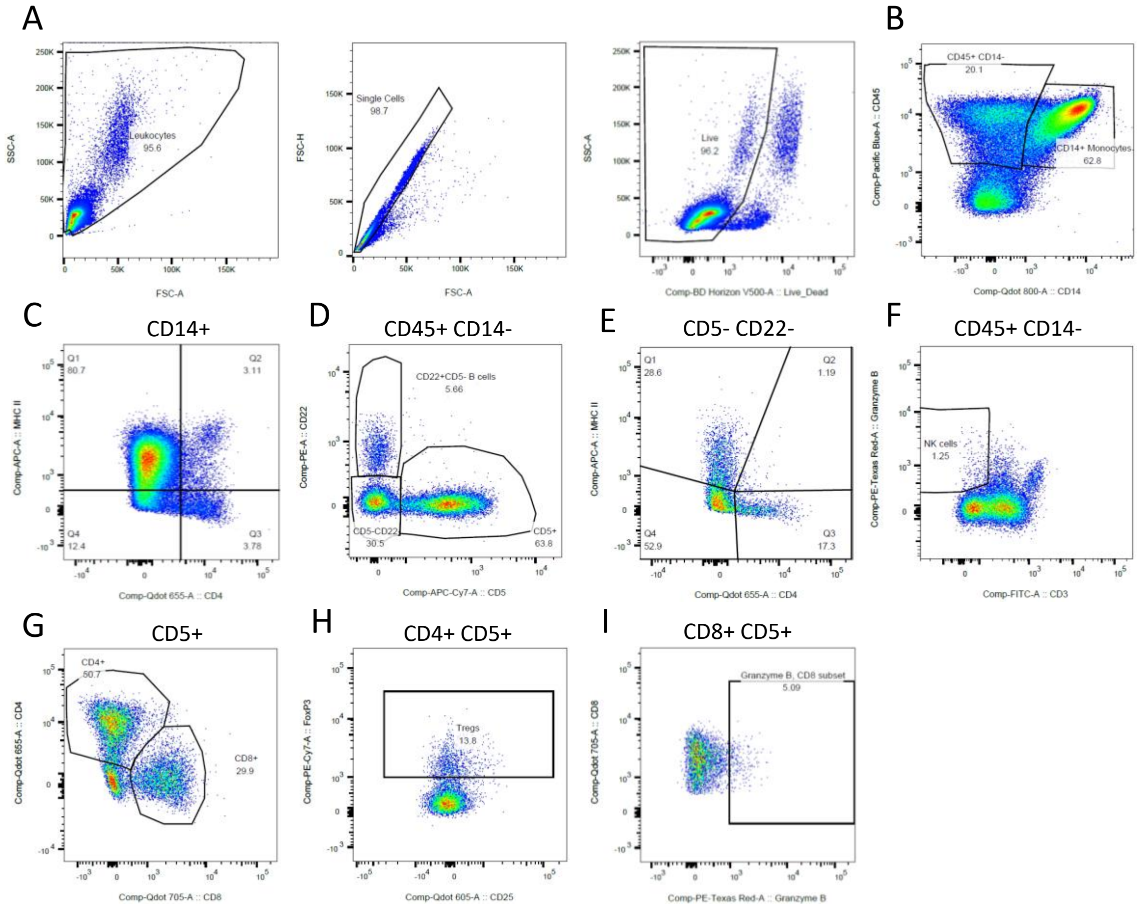

Figure S5. Gating strategy for immunophenotyping of canine PBMCs. (A) Leukocytes (SSC-A/FSC-A) were further defined as single (doublet exclusion FSC-H/FSC-A) live cells (SSC-A/Viability Aqua). (B) Live leukocytes were further discriminated in CD45+ leukocytes and CD14+CD45+ monocytes. (C) Differential expression of MHCII and CD4 on monocytic population. (D) CD45+ leukocytes were divided into lymphocytes (CD22+ B cells and CD5+ T cells) and (E) a CD22-CD5- cell population further characterized into MHCII+ antigen presenting cells and CD4+ neutrophils ([1] and references therein). (F) Gating for GzmB+CD3- NK cells. (G) Identification of CD4+ T helper and CD8+ T cytotoxic cells within the CD5+ T cell population, and FoxP3+ regulatory T cells differentially expressing CD25 within the CD4+ T cell population (H). (I) Expression of cytotoxic cell marker GzmB within the CD8+ T cell population. Parent population is indicated above the plots. Description of the immunophenotype is provided in table S10.

1. Pantelyushin S, Ranninger E, Bettschart-Wolfensberger R, Vom Berg J (2020) OMIP-065: Dog Immunophenotyping and T-Cell Activity Evaluation with a 14-Color Panel. Cytometry A 97 (10):1024-1027. doi:10.1002/cyto.a.24168. Though this one is the original paper describing the CD4+ status: Moore et al. 1992.

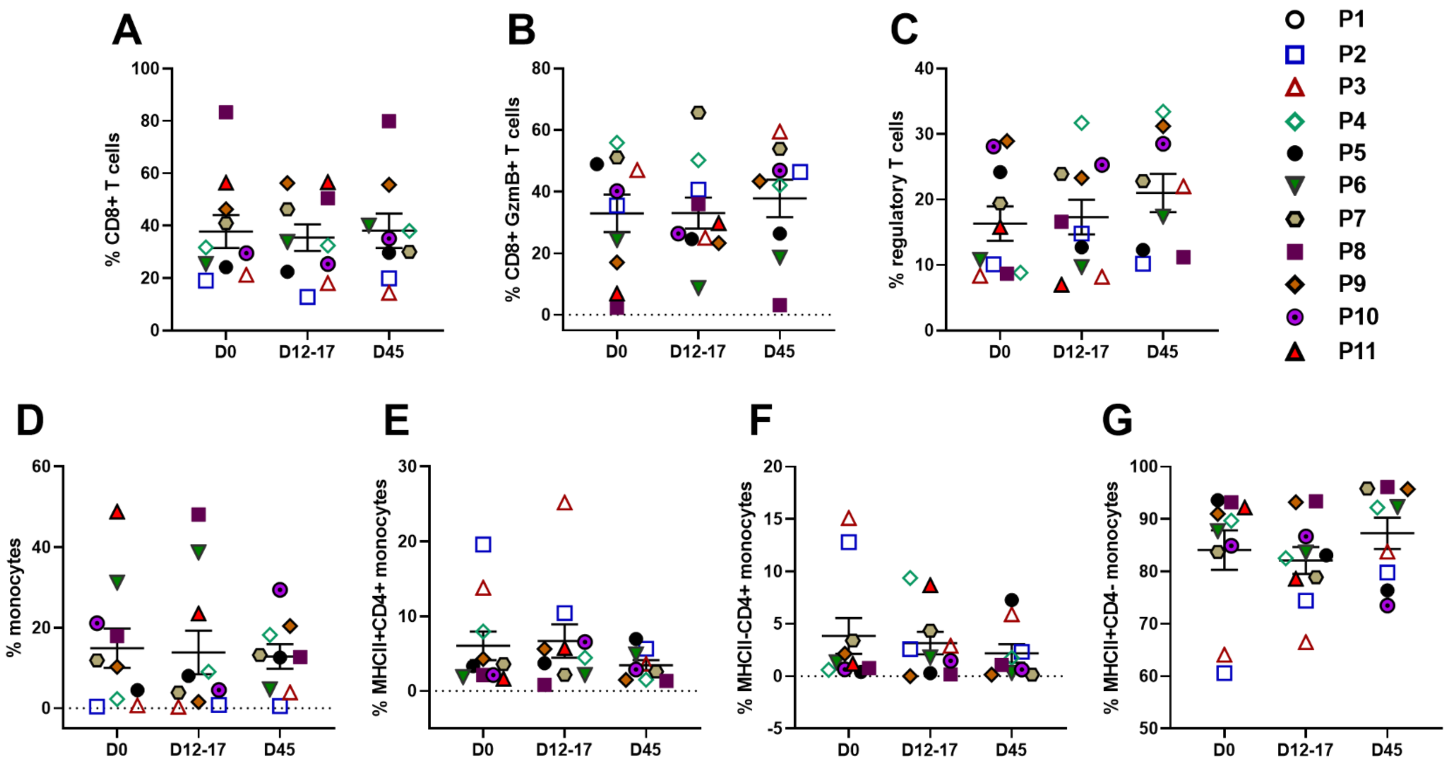

Figure S6. eCPMV-induced changes in blood immune cells. Changes induced by eCPMV treatment of individual CMC patients; $CD8^+$ T cells (A), $CD8^+$Granzyme B^+ T cells (B), regulatory T cells (C), total monocytes (D), $MHCII^+CD4^+$ monocytes (E), $MHCII^-CD4^+$ monotytes (F), and $MHCII^+CD4^-$ monocytes (G). Each companion dog is represented by a colored individual shape as indicated on the right side of C. X-axis indicates the day when measurements were taken. Y-axis indicates the percentages of different immune cells. NR, refers to normal range values.

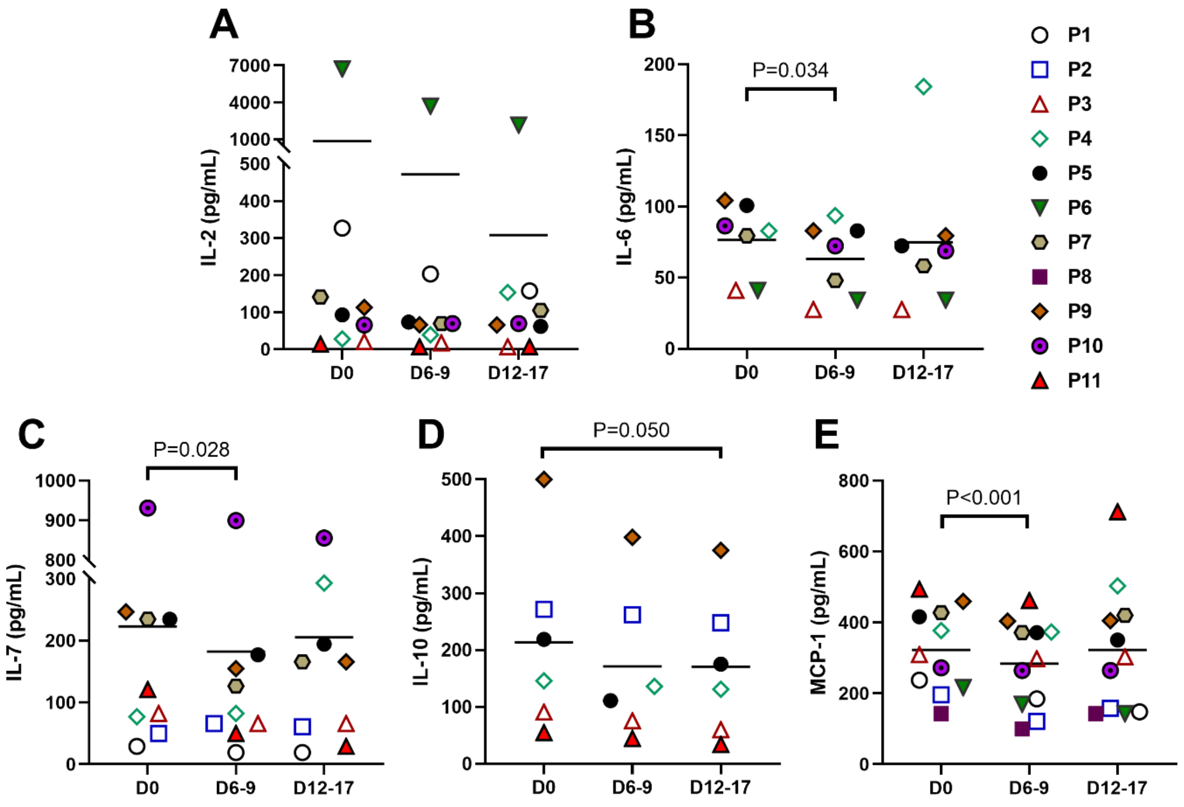

Figure S7. eCPMV immunotherapy induced transitory changes in plasma cytokines in vaccinated dogs. Changes induced by eCPMV injections in (A) IL-2, (B) IL-6, (C) IL-7, (D) MCP-1, and (E) IL-10 plasma levels. Each companion dog is represented by a colored individual shape as indicated on the right side of B. X-axis indicates the day when measurements were taken. Y-axis shows percentage changes in the plasma concentration of different cytokines. P-value obtained by paired Student t-test.

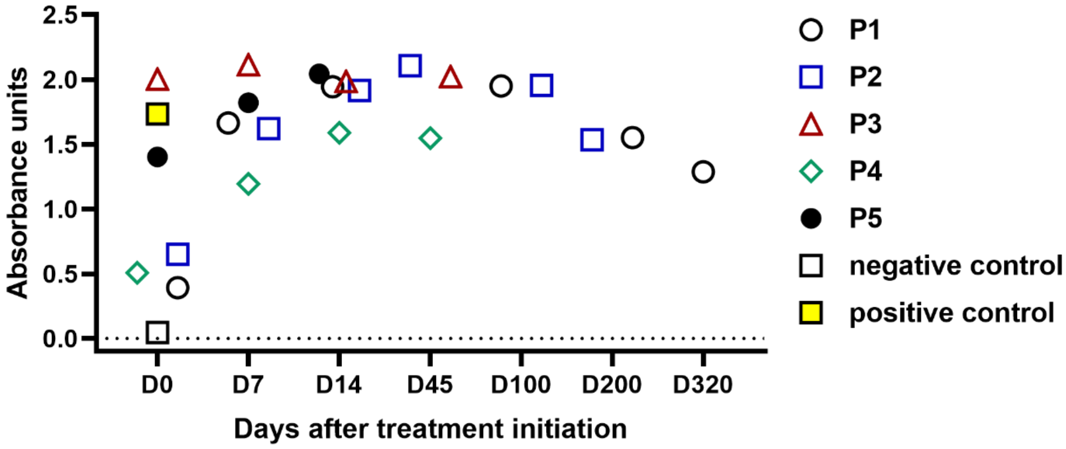

Figure S8. eCPMV immunotherapy induced anti-CPMV antibodies. (A) The levels of anti-CPMV antibodies in five CMC patients increased after injection of eCPMV nanoparticles and remained higher than D0 at various time points. A few dogs were followed beyond surgery day. Each companion dog is represented by a colored individual shape. Absorbance units at 450 nm is on the y-axis and days after eCPMV treatment in the x-axis.

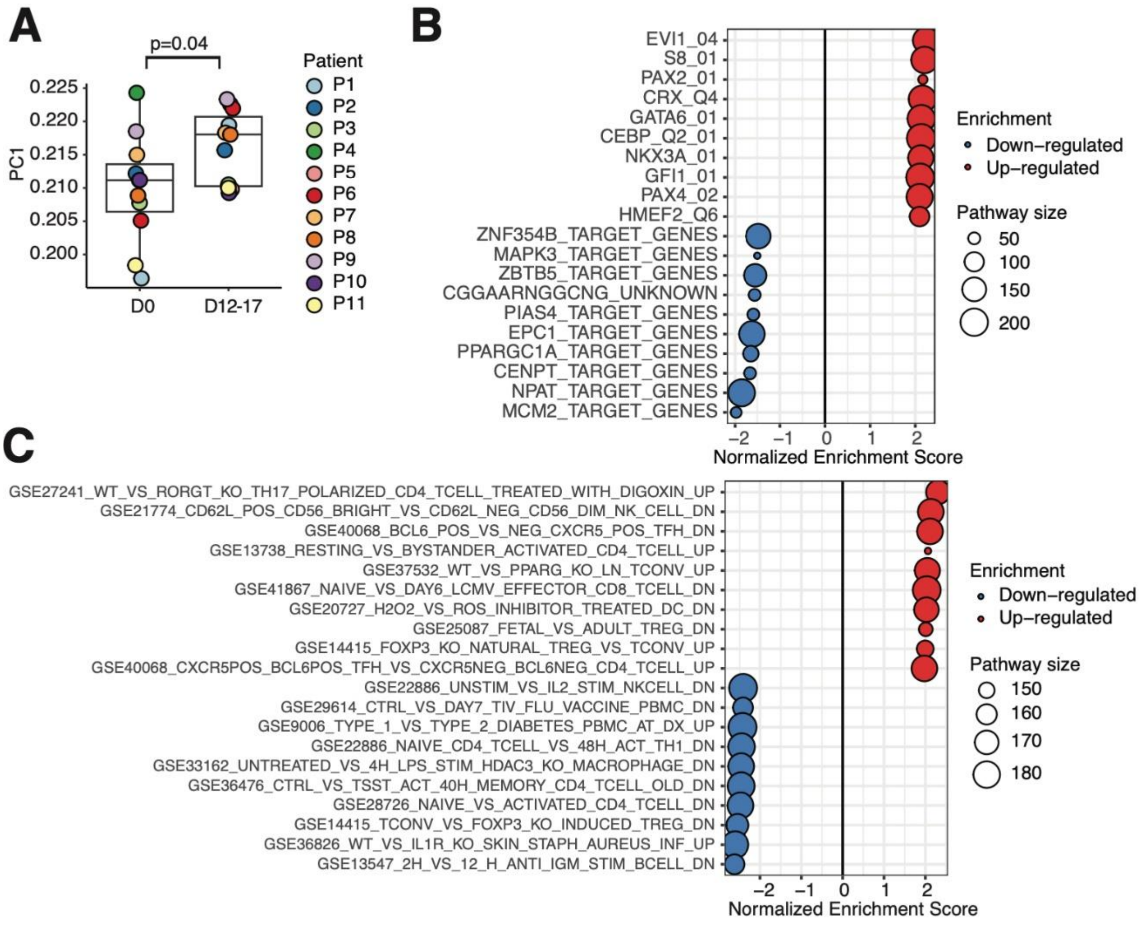

Figure S9. eCPMV immunotherapy induced changes in the TME. (A) Comparison of PC1 values between D0 and D12-17 samples. P-value calculated by unpaired Wilcoxon signed-rank test. (B) Top 10 transcription factor genes significantly upregulated and 10 top transcription factor target genes downregulated on the pathway level by GSEA comparing D12-17 to D0 samples. (C) Top 10 upregulated and 10 downregulated pathways from the immune-related pathway gene set (C7; MSigDB) GSEA comparing D12-17 to D0 samples.

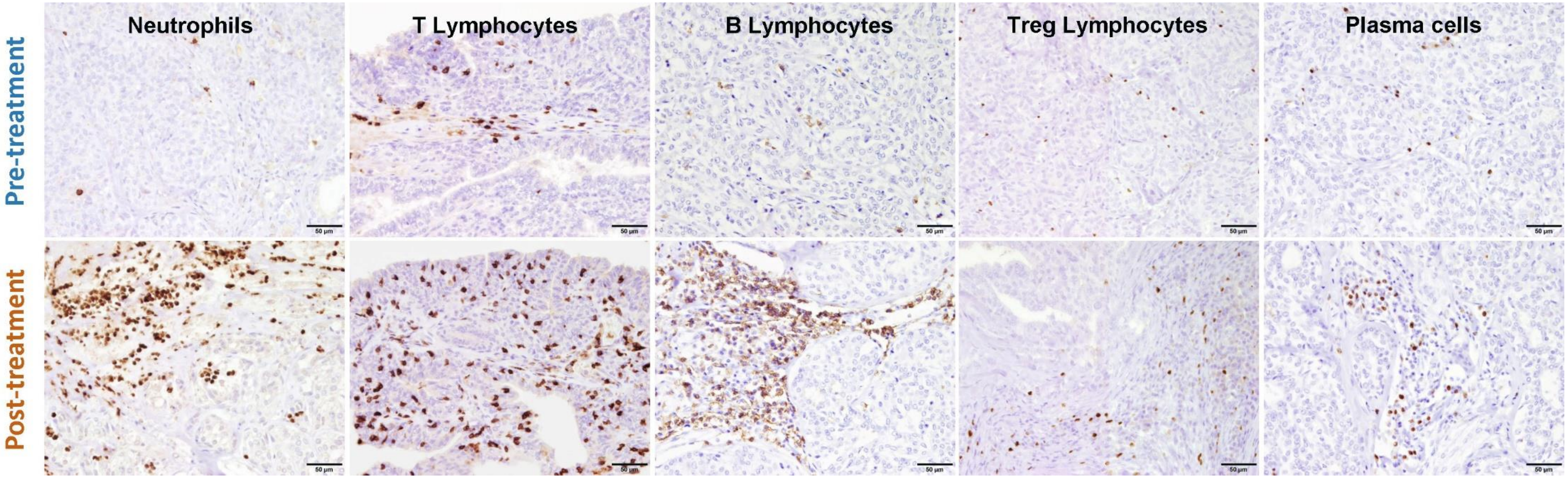

Figure S10. eCPMV immunotherapy increases intratumoral inflammatory cells in patient 7. Representative immunostaining of pre-treatment (D0) and post-treatment (D12-17) tumor tissues. When compared with pre-treatment tumor biopsies, post-treatment tumor biopsies have significant increases in intratumor infiltration with neutrophils (MPO+), T lymphocytes (CD3+), B lymphocytes (CD20+), T regulatory lymphocytes (FoxP3+), and plasma cells (MUM1+). Quantitative analysis is provided in table S11.

Table S1. Breed of eCPMV-treated CMC patients

P1	German Shepherd
P2	Mixed
P3	German Shorthaired Pointer
P4	Miniature Schnauzer
P5	German Shorthaired Pointer
P6	Labrador Retriever
P7	Mixed
P8	German Shepherd
P9	Schnauzer
P10	German Shorthaired Pointer
P11	Yorkshire Terrier

Table S2. List of monoclonal antibodies used for flow cytometry.

Antibody	Clone	Fluorochrome	Provider, cat. #
CD45	YKIX716.13	eFluor 450	eBioscience™, ThermoFisher 48-5450-41
CD25	P4A10	Super Bright 600	eBioscience™, ThermoFisher, 63-0250-42
CD4	YKIX302.9	Super Bright 645	eBioscience™, ThermoFisher, 64-5040-42
CD8a	YCATE55.9	Super Bright 702	eBioscience™, ThermoFisher, 67-5080-42
EOMES	WD1928	PerCP-eFluor 710	eBioscience™, ThermoFisher, 46-48877-42
CD22	RFB-4	PE	ThermoFisher, MHCD2204
FOXP3	FJK-16s	PE-Cyanine7	eBioscience™, ThermoFisher, 25-5773-80
MHC Class II	YKIX334.2	APC	ThermoFisher, 17-5909-42
Ki67	SolA15	Alexa Fluor 700	eBioscience™, ThermoFisher, 56-5698-82
CD5	YKIX322.3	APC-eFluor 780	eBioscience™, ThermoFisher, 47-5050-42
CD14	M5E2	Brilliant Violet 785	Biolegend, 301840
CD3	CA17.2A12	FITC	BioRad, MCA1774F
Granzyme B	Granzyme B	PE-CF594	BD, 562462
Dead cells		Zombie Aqua™ Fixable Viability Kit	Biolegend, 423102

Table S3. List of primary antibodies used for immunohistochemistry.

Antibody	Clone	Antigen Retrieval	Incubation	Concentration	Provider, cat. #
Ki67	SP6	1 mM EDTA, 96ºC, 20 min	90 min, RT.	Ready-to-use	MasterDiagnostica™, MAD-000310QD
ER	OTI3G2	1 mM EDTA, 96ºC, 20 min	15 min, RT.	0.33 µg/ml	Origene™, TA807239
PR	1E2	1 mM EDTA, 96ºC, 20 min	90 min, RT.	Ready-to-use	Ventana Roche™, 790-4296
HER2	Polyclonal	1 mM EDTA, 96ºC, 20 min	120 min, RT.	0.6 µg/ml	Dako™, A0485
MPO	Polyclonal	1 mM EDTA, 96ºC, 20 min	30 min, RT.	33 µg/ml	Dako™, A0398
CD3	Polyclonal	1 mM EDTA, 96ºC, 20 min	120 min, RT.	6 µg/ml	Dako™, A045201
FoxP3	SP97	1 mM EDTA, 96ºC, 20 min	90 min, RT.	Ready-to-use	MasterDiagnostica™, MAD-000536QD
CD20	Polyclonal	1 mM EDTA, 96ºC, 20 min	60 min, RT.	0.33 µg/ml	ThermoFisher™, RB-9013
MUM1	BC-5	1 mM EDTA, 96ºC, 20 min	60 min, RT.	0.2 µg/ml	Biocare™, CRM-352A

Legends: ER, Estrogen Receptor; PR, Progesterone Receptor; HER2, human epidermal growth factor receptor 2; MPO, Myeloperoxidase; RT, room temperature.

Table S4. Tumor changes in target injected lesion in CMC patients by itRECIST criteria.

Patient	Day	Tumor diameter, cm	itRECIST	Response
P1	D0	5.0		
	D8	4.5	-10.0	SD
	D16	4.5	0.0	SD
P2	D0	2.6		
	D7	2.5	-3.8	SD
	D14	2.5	0.0	SD
P3	D0	6.2		
	D7	5.5	-11.3	SD
	D13	5.2	-5.5	SD
P4	D0	3.3		
	D6	3.4	3.0	SD
	D13	3.3	0.0	SD
P5	D0	4.2		
	D9	4.1	-2.4	SD
	D17	3.9	-4.9	SD
P6	D0	6.6		
	D6	6.1	-7.6	SD
	D13	6.1	0.0	SD
P7	D0	2.9		
	D8	2.8	-3.4	SD
	D15	2.5	-10.7	SD
P8	D0	7.5		
	D7	7.1	-5.3	SD
	D12	7.2	1.4	SD
P9	D0	2.7		
	D7	2.4	-11.1	SD
	D13	2.3	-4.2	SD
P10	D0	2.3		
	D6	2.2	-4.3	SD
	D13	2.1	-4.5	SD
P11	D0	5.9		
	D8	4.4	-25.4	SD
	D15	4.3	-2.3	SD

Legends: itRECIST: compared each measurement with previous day of injections; SD, stable disease.

Table S5. Tumor changes in target noninjected lesions in CMC patients by itRECIST criteria.

Days	P1			P2			P3			P5		
	ΔD, cm	δD	Response	ΔD, cm	δD	Response	ΔD, cm	δD	Response	ΔD, cm	δD	Response
**D0**	7.50			1.00			1.20			1.00		
**DTx2**	6.20	-17.33	SD	1.00	0.00	SD	0.80	-33.33	**PR**	1.00	0.00	SD
**DSx**	5.60	-9.68	SD	1.00	0.00	SD	0.80	-33.33	**PR**	0.60	-40.00	**PR**

Days	P7			P8			P9			P11		
	ΔD, cm	δD	Response	ΔD, cm	δD	Response	ΔD, cm	δD	Response	ΔD, cm	δD	Response
**D0**	2.70			7.00			1.00			1.10		
**DTx2**	3.10	14.81	SD	5.50	-21.43	SD	0.90	-10.00	SD	1.10	0.00	SD
**DSx**	2.80	3.70	SD	4.80	-31.43	**PR**	0.90	0.00	SD	1.10	0.00	SD

Legend: ΔD, sum of the maximum diameter of non-injected lesions; δD, percentage of change of the sum of diameters; P1.1, P1.2, etc., location of the untreated tumor in the ipsilateral (I) or contralateral (C) mammary chain; SD, stable disease; PR, partial response; D0, day of first injection; DTx2, day of second injection; DSx, day of surgery.

Applying itRECIST: itRECIST allows a maximum of 5 target-non injected lesions (independent of the organ); mammary lesions of 10 mm or larger were initially selected, and the largest lesions between them were subsequently chosen; if two lesions have the same size, the closer one to the injected tumor was selected to evaluate response to treatment.

Table S6. Tumor changes in nontarget noninjected lesions in CMC patients by itRECIST criteria.

Days	P1			P2			P3			P4			P5			P7			P7			P8		
	D, cm	δD	Resp	D, cm	δD	Resp	D, cm	δD	Resp	D, cm	δD	Resp	D, cm	δD	Resp	D, cm	δD	Resp	D, cm	δD	Resp	D, cm	δD	Resp
	P1.4	C		P2.1	I		P3.1	C		P4.1	C		P5.1	I		P7.2	I		P7.4	I		P8.1	I	
**D0**	0.50			0.50			0.40			0.60			0.50			0.80			0.40			0.50		
**DTx2**	0.50	0.00	Present	0.50	0.00	Present	0.40	0.00	Present	0.60	0.00	Present	0.30	-40.00	Present	0.90	12.50	Present	0.30	-25.00	Present	0.40	-20.00	Present
**DSx**	0.50	0.00	Present	0.30	-40.00	Present	0.40	0.00	Present	0.60	0.00	Present	0.30	-40.00	Present	0.90	12.50	Present	0.30	0.00	Present	0.40	0.00	Present
										P4.2	C					P7.3	I		P7.5	C		P8.2	I	
**D0**										0.60						0.60			0.60			0.50		
**DTx2**										0.60	0.00	Present				0.20	-66.67	Present	0.70	16.67	Present	0.40	-20.00	Present
**DSx**										0.60	0.00	Present				0.20	0.00	Present	0.70	16.67	Present	0.40	0.00	Present

Days	P8			P9			P10			P11			P11			P11			P11			P11		
	D, cm	δD	Resp	D, cm	δD	Resp	D, cm	δD	Resp	D, cm	δD	Resp	D, cm	δD	Resp	D, cm	δD	Resp	D, cm	δD	Resp	D, cm	δD	Resp
	P8.3	I		P9.1	I		P10.1	I		P11.1	I		P11.4	C		P11.6	C		P11.8	C		P11.10	C	
**D0**	0.90			0.40			0.80			0.30			0.70			0.50			0.40			0.50		
**DTx2**	0.70	-22.22	Present	0.40	0.00	Present	0.70	-12.50	Present	0.30	0.00	Present	0.60	-14.29	Present	0.40	-20.00	Present	0.40	0.00	Present	0.40	-20.00	Present
**DSx**	0.70	0.00	Present	0.40	0.00	Present	0.70	0.00	Present	0.30	0.00	Present	0.60	0.00	Present	0.40	0.00	Present	0.50	25.00	Present	0.40	0.00	Present
				P9.2	I					P11.2	I		P11.5	C		P11.7	C		P11.9	C		P11.11	C	
**D0**				0.70						0.40			0.50			0.20			0.40			0.70		
**DTx2**				0.70	0.00	Present				0.50	25.00	Present	0.50	0.00	Present	0.30	50.00	Present	0.30	-25.00	Present	0.70	0.00	Present
**DSx**				0.70	0.00	Present				0.40	0.00	Present	0.40	-20.00	Present	0.20	0.00	Present	0.50	66.67	Present	0.70	0.00	Present

Legend: δD, percentage of change of the diameter (D); D, maximum diameter of non-injected lesion; Resp, response based on itRECIST; D0, day of first injection; DTx2, day of second injection; DSx, day of surgery; P1.4, P7.4, etc., location of the untreated tumor in the ipsilateral (I) or contralateral (C) mammary chain.

Applying itRECIST: itRECIST allows a maximum of 5 target noninjected lesions (independent of the organ) with a minimum size of 10 mm; other lesions are defined as nontarget noninjected and are included in this table. The response of nontarget lesions is defined as absent, present, or unequivocal progression.

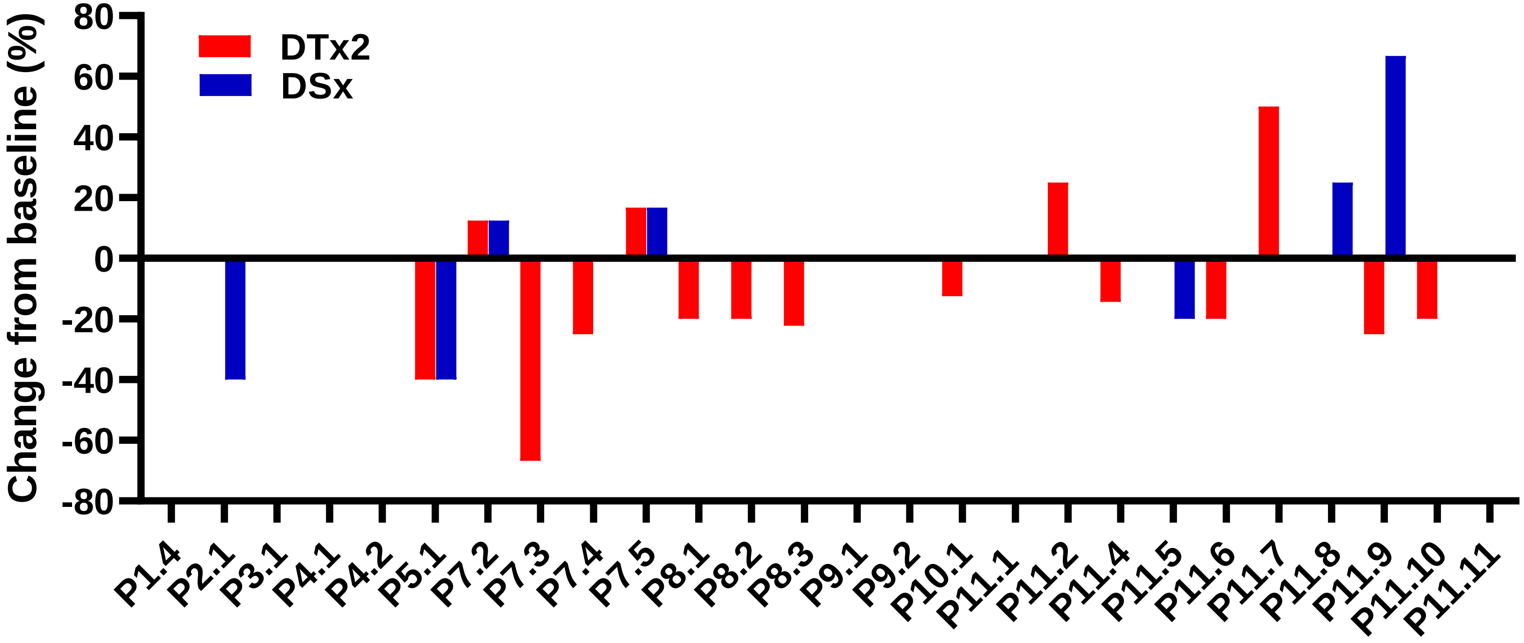

Figure to table S6. Evaluation of the changes in the non-target noninjected lesions based on itRECIST. DTx2, day of second injection; DSx, day of surgery.

Table S7. Blood cell and biochemistry changes during eCPMV immunotherapy in CMC patients

Variable	D0	DTx2	DSx	D45	P value*
Hematocrit (%)	45.2 ± 2.1	42.8 ± 2.2	41.3 ± 2.4	48.1 ± 1.4	0.067; 0.052; 0.467; 0.196
Red cells (cells x 10^6/μL)	6.6 ± 0.3	6.4 ± 0.3	6.1 ± 0.4	7.2 ± 0.3	0.260; 0.071; 0.347; 0.126
Hemoglobin (g/dL)	15.1 ± 0.6	14.6 ± 0.7	14.0 ± 0.8	16.4 ± 0.4	0.193; 0.094; 0.362; **0.033**
Platelets (cells x 10^3/μL)	350.9 ± 37.6	387.6 ± 27.6	366.1 ± 37.5	327.8 ± 44.5	0.182; 0.341; 0.328; 0.693
Lymphocytes (cells/μL)	2412.8 ± 351.3	2262.0 ± 204.5	2104.0 ± 239.6	2280.3 ± 440.3	0.929; 0.477; 0.541; 0.374
Monocytes (cells/μL)	596.1 ± 113.0	569.7 ± 113.2	592.6 ± 116.9	390.2 ± 69.1	0.807; 0.968; 0.848; 0.170
Mature neutrophils (cells/μL)	6977.8 ± 864.9	8373.6 ± 1146.7	7848.4 ± 1020.0	6155.1 ± 420.7	**0.022**; 0.262; 0.549; 0.296
Immature neutrophils (cells/μL)	35.5 ± 24.6	219.9 ± 89.5	167.3 ± 61.4	30.2 ± 22.5	**0.046**; 0.110; 0.515; 0.715
Neutrophils to lymphocytes ratio	3.1 ± 0.3	4.4 ± 1.1	3.8 ± 0.3	3.2 ± 0.3	0.374; 0.110; 0.608; 0.964
Albumin (g/dL)	3.0 ± 0.2	3.1 ± 0.2	3.0 ± 0.2	2.8 ± 0.1	0.542; 0.892; 0.564; 0.611
Total proteins (g/dL)	7.1 ± 0.2	7.3 ± 0.2	7.1 ± 0.3	7.0 ± 0.2	**0.044**; 0.652; 0.179; 0.374
Total globulins (g/dL)	4.1 ± 0.4	4.5 ± 0.2	4.8 ± 0.2	4.5 ± 0.3	0.296; 0.131; **0.002**; 0.211
Glucose (mg/dL)	103.6 ± 5.7	109.3 ± 3.8	105.6 ± 5.2	108.0 ± 2.4	0.348; 0.741; 0.565; 0.445
Urea (mg/dL)	40.2 ± 10.3	38.6 ± 5.0	48.4 ± 10.8	42.1 ± 6.4	0.859; 0.055; 0.168; 0.450
Creatinine (mg/dL)	0.9 ± 0.1	0.9 ± 0.1	0.9 ± 0.1	1.0 ± 0.1	0.794; 0.718; 0.811; 0.188
ALT (U/L)	37.0 ± 9.1	27.6 ± 3.7	32.6 ± 6.7	28.6 ± 3.4	0.138; 0.878; 0.341; 0.476

Legends: D0, day of first injection, DTx2 day of second injection, and DSx, day of surgery; ± denotes standard error; *, P values estimated by Student t-test or Wilcoxon test for DTx2 and DSx compared to D0, first and second values; between DTx2 and DSx, third value; and between D0 and 30 days (D45) after surgery, fourth value.

Table S8. eCPMV-induced changes in peripheral PMBCs.

Cell type	Markers*	D0	DSx	D45	P value**
T cells	CD14- CD5+ CD22-	84.0 ± 3.3	79.5 ± 3.9	78.6 ± 4.7	0.508; 0.173; 0.380
CD8+ T cells	CD14- CD5+ CD22- CD4- CD8+	37.8 ± 6.3	35.5 ± 5.1	38.1 ± 6.6	0.550; 0.418; 0.258
CD8+ GzmB+ T cells	CD14- CD5+ CD22- CD4- CD8+ GZMB+	32.9 ± 6.1	33.0 ± 5.1	37.8 ± 6.0	0.984; 0.691; 0.530
CD4+ T cells	CD14- CD5+ CD22- CD4+ CD8-	39.7 ± 3.9	42.1 ± 3.5	37.7 ± 4.3	0.575; 0.256; 0.065
Regulatory T cells	CD14- CD5+ CD22- CD4+ CD8- FoxP3+	16.3 ± 2.6	17.3 ± 2.6	21.0 ± 2.9	0.755; 0.204; 0.263
Treg+/CD8+ ratio		0.5 ± 0.1	0.6 ± 0.1	0.7 ± 0.1	0.510; 0.415; 0.822
Natural killer cells	CD14- CD3- GZMB+	1.5 ± 0.4	0.9 ± 0.2	1.8 ± 0.6	0.194; 0.785; 0.229
B cells	CD14- CD5- CD22+	6.0 ± 2.0	5.8 ± 1.9	5.0 ± 1.4	0.857; 0.773; 0.631
Antigen presenting cells	CD14- CD5- CD22- CD4- MHC+	70.1 ± 5.8	56.9 ± 7.4	70.0 ± 5.4	0.189; 0.905; 0.351
Neutrophils	CD14- CD5- CD22- CD4+ MHC-	4.7 ± 1.9	11.9 ± 7.3	1.5 ± 0.5	0.799; 0.142; 0.515
Monocytes	CD14+	14.9 ± 4.9	13.8 ± 5.4	12.9 ± 3.0	0.721; 0.687; 0.594
$MHCII^{+}CD4^{+}$ monocytes (Mo1)	CD14+ MHC+ CD4+	6.0 ± 1.9	6.7 ± 2.2	3.4 ± 0.7	0.721; 0.214; 0.173
$MHCII^{-}CD4^{+}$ monocytes (Mo2)	CD14+ MHC- CD4+	3.8 ± 1.7	3.2 ± 1.1	2.2 ± 0.9	0.878; 0.260; 0.793
$MHCII^{+}CD4^{-}$ monocytes (Mo3)	CD14+ MHC+ CD4-	84.1 ± 3.8	82.1 ± 2.6	87.3 ± 3.0	0.445; 0.214; 0.189

Legends: *, gating was performed on CD45+ as illustrated in figure S5; ± denotes standard error; **, P values estimated by Student t-test or Wilcoxon test for DSx vs. D0, first value; D45 (30 days after surgery) vs. D0, second value; and D45 vs. D0, third value; MHCII, major histocompatibility complex type II; GzmB, Granzyme B.

Table S9. eCPMV-induced changes in plasma cytokines levels.

Variable	D0	DTx2	DSx	P value*
GM-CSF (pg/mL)	160.0 ± 42.7	144.8 ± 45.9	176.9 ± 49.8	0.057; 0.519; 0.218
IFN-g (pg/mL)	65.5 ± 25.2	67.5 ± 21.1	67.2 ± 22.9	0.577; 0.290; 0.954
KC-like (pg/mL)	32.8 ± 15.9	189.2 ± 149.7	81.5 ± 44.2	0.477; 0.213; 0.477
IP-10 (pg/mL)	271.2 ± 260.6	209.4 ± 200.3	143.9 ± 135.8	0.109; 0.109; 0.109
IL-2 (pg/mL)	836.2 ± 736.6	425.0 ± 364.1	278.1 ± 208.4	0.051; 0.086; 0.499
IL-6 (pg/mL)	70.5 ± 10.5	59.5 ± 9.3	75.0 ± 19.6	**0.034**; 0.237; 0.917
IL-7 (pg/mL)	222.8 ± 92.9	182.3 ± 91.3	205.3 ± 86.6	**0.028**; 0.173; 0.612
IL-8 (pg/mL)	2121.0 ± 410.6	2966.0 ± 545.3	2872.0 ± 770.8	0.223; 0.356; 0.930
IL-10 (pg/mL)	213.6 ± 65.9	171.2 ± 54.7	170.5 ± 51.7	0.084; **0.050**; 0.962
IL-15 (pg/mL)	182.1 ± 48.7	163.7 ± 51.8	160.6 ± 41.0	0.291; 0.339; 0.698
IL-18 (pg/mL)	110.2 ± 31.6	84.1 ± 26.5	95.8 ± 26.4	0.062; 0.477; 0.107
MCP-1 (pg/mL)	322.6 ± 35.7	283.5 ± 37.3	322.7 ± 54.5	**<0.001**; 0.997; 0.153
TNFa (pg/mL)	42.2 ± 15.2	37.6 ± 15.1	50.2 ± 12.7	0.225; 0.285; 1.000

Legends: ± denotes standard error; *, P values estimated by Student t-test or Wilcoxon test. First value, DTx2 vs. D0; second value, DSx vs. D0; third value, DTx2 vs. DSx.

Table S10. DEGs comparing D12-17 to D0. Genes with FRD<0.25 are included.

Gene	baseMean	log2FoldChange	lfcSE	stat	pvalue	padj	Dog gene	Human gene
ENSCAFG00000019755	2509.134579	7.525472365	1.390146003	5.41344028	6.18E-08	0.000896528	LOC491264	
ENSCAFG00000056053	402.9577443	3.666904011	0.725919016	5.051395442	4.39E-07	0.003180026	GAS1	
ENSCAFG00000032096	20.45163266	5.639170121	1.142440887	4.936071691	7.97E-07	0.003853003	SPINT3	SPINT3
ENSCAFG00000011769	19.95695456	3.05369952	0.656996691	4.647967884	3.35E-06	0.012152603	ANK2	ANK2
ENSCAFG00000050188	12.4059289	4.315144886	0.951497628	4.535108402	5.76E-06	0.016697585	NA	NA
ENSCAFG00000008658	50.25558426	5.78871536	1.290102785	4.48701873	7.22E-06	0.017044286	NA	NA
ENSCAFG00000012424	19.44082679	3.180792521	0.717317788	4.43428641	9.24E-06	0.017044286	SYNPO2	SYNPO2
ENSCAFG00000031894	14.7231178	3.670153104	0.828390409	4.430463055	9.40E-06	0.017044286	TENT5A	TENT5A
ENSCAFG00000050279	68.95609874	0.918752079	0.20892961	4.397423987	1.10E-05	0.017649849	NA	NA
ENSCAFG00000015536	14.33979012	3.311105545	0.798520449	4.146550722	3.38E-05	0.048943983	NAALADL2	NAALADL2
ENSCAFG00000021436	65.91728489	2.740208802	0.674937064	4.059947136	4.91E-05	0.064705871	U5	
ENSCAFG00000015324	233.2870096	2.114635773	0.528326392	4.002517773	6.27E-05	0.075733839	TNFAIP8L3	TNFAIP8L3
ENSCAFG00000018598	6.111420663	3.524534237	0.895925021	3.933961162	8.36E-05	0.093204944	C6	C6
ENSCAFG00000004733	28.5806228	3.349011331	0.863648263	3.877749167	0.000105427	0.098383892	DGKH	DGKH
ENSCAFG00000008586	137.1071192	2.184537657	0.563581414	3.876170512	0.000106113	0.098383892	ADAMTS1	ADAMTS1
ENSCAFG00000003476	96.55137156	-1.277971365	0.330234896	-3.869885893	0.000108886	0.098383892	BLVRA	BLVRA
ENSCAFG00000048887	33.28290173	3.972138043	1.030164802	3.855827762	0.000115339	0.098383892	LOC475936	
ENSCAFG00000001460	17.55543804	4.00182543	1.053375574	3.799049007	0.000145252	0.112062655	PTPRD	PTPRD
ENSCAFG00000008948	2163.11334	3.290357842	0.869349646	3.78484981	0.000153801	0.112062655	LYZF2	
ENSCAFG00000012156	7.82735837	3.269281582	0.864059897	3.783628419	0.000154559	0.112062655	ARSJ	ARSJ
ENSCAFG00000010877	241.4431011	2.53397383	0.674632748	3.756078902	0.000172596	0.119181854	ABCA9	ABCA9
ENSCAFG00000020357	84.17780787	1.659490744	0.443482216	3.741955559	0.000182594	0.120354221	DNAJB4	DNAJB4
ENSCAFG00000056420	143.448878	0.658418598	0.176769438	3.724730959	0.000195524	0.122091281	NA	NA
ENSCAFG00000057505	6.687626415	5.542990485	1.49662586	3.703658097	0.000212513	0.122091281	NA	NA
ENSCAFG00000005886	483.494185	1.036263917	0.280035728	3.700470375	0.0002152	0.122091281	CRIM1	CRIM1
ENSCAFG00000018828	15.1543832	3.320539855	0.900759857	3.686376374	0.00022747	0.122091281	CIDEA	CIDEA
ENSCAFG00000028240	27.53725267	2.59504787	0.705068462	3.68056155	0.000232721	0.122091281	Y_RNA	
ENSCAFG00000020340	61.65535072	2.613472963	0.710710683	3.677267032	0.000235746	0.122091281	ADGRL4	ADGRL4
ENSCAFG00000010916	32.73544747	2.058036981	0.562864046	3.656366039	0.000255816	0.127916821	GSTCD	GSTCD
ENSCAFG00000051636	7.218969663	4.110151646	1.135648518	3.619211033	0.000295503	0.1428361	AADAC	AADAC
ENSCAFG00000023943	8.196899822	2.731890784	0.760092508	3.594155651	0.000325445	0.152234888	ZNF565	ZNF565
ENSCAFG00000057909	24.69197299	2.32277069	0.655241528	3.54490763	0.000392751	0.177977432	NA	NA
ENSCAFG00000012433	50.59760269	1.507778907	0.427679549	3.525487508	0.000422704	0.18549994	PER2	PER2
ENSCAFG00000053795	7.612253584	3.030614943	0.863498989	3.509691361	0.000448627	0.18549994	NA	NA

ENSCAFG00000058871	38.20545848	0.762637555	0.217685385	3.503393461	0.00045937	0.18549994	NA	NA
ENSCAFG00000013796	7.376184239	2.363299253	0.674702515	3.502727798	0.00046052	0.18549994	NA	NA
ENSCAFG00000017197	5.116611976	4.006060031	1.147443315	3.491292319	0.00048069	0.18839151	ATP8B2	ATP8B2
ENSCAFG00000004443	96.58930524	2.728305472	0.783858654	3.480608982	0.000500275	0.190907669	FOSB	FOSB
ENSCAFG00000013655	170.410236	1.712852379	0.49846468	3.436256261	0.000589813	0.214094385	HMCN1	HMCN1
ENSCAFG00000014549	27.51041817	1.952575426	0.569045868	3.431314656	0.000600663	0.214094385	KLHL20	KLHL20
ENSCAFG00000019986	72.08752414	-1.288764733	0.37581911	-3.429215547	0.000605329	0.214094385	NTMT1	NTMT1
ENSCAFG00000019601	23.86798921	2.311437652	0.676394974	3.417289811	0.000632479	0.218370945	SOX8	SOX8
ENSCAFG00000056756	269.2213059	1.357040915	0.401126306	3.38307634	0.000716787	0.241723929	NA	NA
ENSCAFG00000012440	66.50128474	1.883226457	0.55784107	3.375919342	0.000735695	0.24246161	SLC16A9	SLC16A9
ENSCAFG00000012521	188.232897	0.824241854	0.244706977	3.368280974	0.000756385	0.243740764	TLK1	TLK1
ENSCAFG00000001612	81.03427653	1.069485296	0.319755972	3.344692168	0.000823739	0.246644321	DENND4C	DENND4C
ENSCAFG00000051148	24.91054124	1.555073728	0.464950166	3.344603018	0.000824004	0.246644321	NA	NA
ENSCAFG00000053543	3.581715154	4.03236792	1.207544666	3.339311608	0.000839863	0.246644321	NA	NA
ENSCAFG00000007950	898.2296987	1.762268791	0.5290095	3.331261139	0.000864535	0.246644321	MAP1B	MAP1B
ENSCAFG00000013309	121.8907128	1.60177089	0.481772356	3.324746367	0.00088499	0.246644321	INO80D	INO80D
ENSCAFG00000029981	97.51635545	-2.729069479	0.821488526	-3.32210298	0.000893417	0.246644321	NA	NA
ENSCAFG00000015682	8.645777145	-2.596136251	0.783056286	-3.31538907	0.000915157	0.246644321	LIPJ	LIPJ
ENSCAFG00000006864	44.33336954	1.515366696	0.457357706	3.313307449	0.000921996	0.246644321	MICU3	MICU3
ENSCAFG00000017841	1199.595636	0.422067043	0.127535334	3.309412607	0.000934919	0.246644321	G3BP1	G3BP1
ENSCAFG00000012637	139.0713722	-1.009608484	0.305087356	-3.309243944	0.000935483	0.246644321	C28H10orf88	C10orf88

Table S11 Immunohistochemistry changes induced by eCPMV immunotherapy in injected tumors.

Variable	D0	DSx	Fold-change*	P value+
Neutrophils (MPO+ cells/mm^2)	46.9 ± 13.6	294.1 ± 91.2	6.3	**0.020**
T lymphocytes (CD3+ cells/mm^2)	49.0 ± 12.8	217.9 ± 73.0	4.4	**0.006**
B lymphocytes (CD20+ cells/mm^2)	64.8 ± 16.9	222.0 ± 77.3	3.4	**0.016**
Treg lymphocytes (FoxP3+ cells/mm^2)	15.9 ± 4.0	45.3 ± 14.8	2.8	**0.016**
Plasma cells (MUM1+ cells/mm^2)	86.6 ± 33.5	226.8 ± 41.5	2.6	**0.021**
FoxP3+/CD3+ ratio	0.38 ± 0.07	0.20 ± 0.02		**0.003**
Ki67 PI (%)	12.5 ± 1.7	17.0 ± 3.6	1.4	0.260

Legends: *, Fold-change relative to D0; ± denotes standard error; +, P values estimated by Student t-test or Wilcoxon test for DSx (D12-17) compared to D0; MPO, myeloperoxidase; Treg, T regulatory lymphocytes; PI, proliferation index.
